# Setting research priorities for global respiratory medicine within the National Institute for Health Research (NIHR) Global Health Research Unit in Respiratory Health (RESPIRE)

**DOI:** 10.7189/jogh.08.020314

**Published:** 2018-12

**Authors:** Igor Rudan, Dhiraj Agrawal, Norita Hussein, Ai Theng Cheong, Steve Cunningham, David Dockerell, Sazlina Shariff Ghazali, Deesha Ghorpade, Monsur Habib, Tabish Hazir, Sanjay Juvekar, Anand Kawade, Ping Yein Lee, Su May Liew, Saturnino Luz, Ee Ming Khoo, Harish Nair, John Norrie, Rutuja Patil, Hilary Pinnock, Siti Nurkamilla Ramdzan, Sudipto Roy, Hani Salim, Pam Smith, Hana Mahmood Yahya, Siân Williams, Harry Campbell, Aziz Sheikh

**Affiliations:** 1 Usher Institute, The University of Edinburgh, Edinburgh, UK; 2 Vadu Rural Health Program, King Edward Memorial Hospital Research Centre (KEMHRC) Pune, India; 3 Department of Primary Care Medicine, Faculty of Medicine, University of Malaya, Malaysia; 4 Department of Family Medicine, University Putra, Malaysia; 5 Child Life and Health, The University of Edinburgh, Edinburgh, UK; 6 MRC Centre for Inflammation Research, The University of Edinburgh, Edinburgh, UK; 7 Chest Research Foundation, India; 8 Bangladesh Primary Care Respiratory Society; 9 Pakistan Institute of Medical Sciences; 10 Maternal Neonatal and Child Health Research Network (MNCHRN), Pakistan; 11 Nursing Edinburgh, Edinburgh, UK; 12 International Primary Care Respiratory Group, Edinburgh, UK

The NIHR Global Health Research Unit in Respiratory Health (henceforth ‘RESPIRE’) is a research and academic capacity development initiative funded by the UK Government through its National Institute of Health Research (NIHR). RESPIRE’s focus is to undertake applied respiratory health in both communicable and non-communicable disorders, which has the potential to improve the respiratory health of children and adults in Bangladesh, India, Malaysia and Pakistan [1]. RESPIRE’s working ethos is to work bottom-up to undertake research priorities that have been identified by RESPIRE investigators in our partner low- and middle-income countries (LMICs) [1]. To this end, we conducted an internal process of identifying research priorities within the RESPIRE collaboration using the Child Health and Nutrition Research Initiative's (CHNRI) method [2]. The outcomes of this process were then used, in conjunction with other approaches, to develop the list of the research projects led by RESPIRE investigators that would receive financial support from the RESPIRE budget [2].

## THE CHNRI EXERCISE TO IDENTIFY RESEARCH PRIORITIES WITHIN RESPIRE

We first asked investigators from the RESPIRE collaboration to submit their ideas for research projects and PhD projects, expecting that those ideas would mainly be generated within Bangladesh, India, Malaysia and Pakistan, in line with their local needs. We then conducted an exercise in assessment of all submitted ideas using the CHNRI method, described briefly in the **Box 1** [3-11]. A total of 35 investigators who were named on the RESPIRE research grant proposal submitted 37 research ideas. We then prepared those ideas for scoring and invited both the 35 proposers and a further 30 members from their academic teams to take part in the process of scoring ideas. The scoring was based on 10 criteria, which were agreed in advance by the RESPIRE team during their first meeting in Edinburgh in June 2017. These criteria were:

BOX 1 **The CHNRI method for setting research priorities**

The CHNRI method uses the principle of crowdsourcing to score proposed research ideas against a pre-defined set of criteria. This enables funders and policymakers to view the strengths, the weaknesses and relative ranking of each proposed research idea, based on submitted opinions of a larger number of experts. This method uses a systematic, transparent, and democratic approach to priority setting. While it allows researchers to independently generate and score research questions (RQs), it also involves funders, policymakers, and other stakeholders at an early stage of the process, ensuring their ownership in the outcomes. The CHNRI method has thus far been implemented in about 100 studies led by multilateral organisations (eg, World Health Organization, United Nations International Children's Emergency Fund (UNICEF)), national governments (eg, China, India, Iran, South Africa), and funders (eg, The Bill and Melinda Gates Foundation) to set research priorities in areas ranging from the reduction of global child mortality, dementia, or disability to the efficient execution of national health plans. The recognised advantages of this method include its systematic nature, transparency and replicability, clearly defined context and criteria, involvement of the funders, stakeholders and policy makers, a structured way of obtaining information, informative and intuitive quantitative outputs, studying the level of agreement over each proposed research idea, and independent scoring of many experts, thus limiting the influence of individuals on the rest of the group [2-11].

1. Answerability: is this research question likely to be answered by this research using the proposed methods and approaches?
2. Feasibility: is this research question likely to lead to deliverable outcomes over the time scale of this project?
3. Effectiveness: is this research question likely to lead to interventions that will effectively reduce disease burden, change provision of care, change policy or practice?
4. Applicability: is this research question translational in nature, in accordance to RESPIRE's overall aims and objectives?
5. Affordability: is this research question affordable within the context and a good value for money in the way it is proposed?
6. Potential for cross-country scalability: is this research question scalable to other populations, providing an opportunity to be conducted/adapted in several of our partner countries?
7. Burden size: does this research question address a significant healthcare problem and/or disease burden?
8. Equity: is this research question likely to reduce inequity?
9. Safety: is this research question likely to have any harmful or unintended consequences?
10. Sustainability: would this research question create data or resources that will lead to opportunities for further, sustainable funding?

The contextual background to guide the scoring was defined in terms of space, time, population of interest and disease burden of interest, as is standard practice in the CHNRI process [3,4,11]. Space was defined as the four partner countries (ie, Bangladesh, India, Malaysia and Pakistan), the time as the interval between now and year 2025, the population of interest was defined as respiratory disease sufferers, and the disease burden of interest was defined as “all respiratory diseases” within the defined space, time and population.

The scorers were also instructed to think beyond the endpoints of research questions and to keep in mind their broader scope and relevance. Assessing some of the proposed research questions was only possible if thinking was shifted from purely assessing the likelihood that the proposed research would achieve their endpoints, to what those endpoints could mean in the wider context and how these could help improve the overall current situation in terms of risk avoidance and intervention coverage at the level of the four partner countries in 2018.

A total of 26 RESPIRE researchers (among the 65 invited) returned their scores by the stated deadline and this allowed us to conduct the analysis of their input, which was performed in line with the guidelines for implementation of the CHNRI process [11]. The scorers were asked to assess each proposed research idea according to the 10 questions posed above as “yes” (coded as 1 point), “no” (0 points), “not sure” (0.5 points) or “don't know” (input left blank). The received scores allowed computation of “research priority scores” for each criterion and the overall priority score, the latter being used for the final ranking of the proposed research questions (**Table 1**) [3,4,11]. In addition, the “average expert agreement” (AEA) was computed for each proposed research question to demonstrate the level of controversy related to each proposed research question among the scorers who took part in the CHNRI exercise [3,4,11].

**Table 1:** The final rank of 37 proposed research questions that relate to global respiratory health and that could be conducted within the RESPIRE programme*

Rank	Proposed research question	Question number	Answerable?	Feasible?	Effective?	Applicable?	Affordable?	Scalable?	Burden size?	Equitable?	Safe?	Sustainable?	Overall Score (unweighed)	Average Expert Agreement
1	Measuring the burden of COPD in the four partner countries	15	94	93	83	85	93	96	98	92	95	91	91.9	0.804
2	HEAL-ASTHMA (Phase 1B): Quasi-experimental feasibility study of a pictorial action plan for asthma in Malaysia	21	95	92	90	93	95	88	95	85	95	75	90.2	0.683
3	Implementation of pulse oximetry as a point-of-care diagnostic in IMCI health facilities	32	93	98	93	89	81	83	93	82	83	93	88.6	0.665
4	Development of a protocol for a COPD prevalence study in India, Bangladesh, Pakistan and Malaysia	2	95	91	75	76	88	96	96	84	91	93	88.6	0.726
5	Assessing the feasibility of using a well-established teleconsultation facility (Micro-Health Centre -MHC) in management of COPD and Asthma in a resource constrained remote rural area	12	90	93	91	92	79	79	96	91	85	89	88.3	0.709
6	Perception of under-five pneumonia among caregivers of selected rural and semi urban communities of Pakistan: A mixed methodology-based study	31	88	98	83	76	89	83	98	87	95	83	88.0	0.674
7	HEAL-ASTHMA (Phase 1A): A cross-sectional study to determine the prevalence of limited health literacy	20	95	95	83	73	92	83	100	93	92	67	87.3	0.674
8	Haze project in Malaysia: reporting haze and studying respiratory effects	24	81	88	92	92	86	73	96	91	92	79	87.0	0.404
9	Developing and piloting an ICT-based intervention for adult asthma with limited health literacy to improve asthma self-management	7	98	82	83	94	86	80	98	78	89	82	86.9	0.683
10	Setting up a registry for asthma to measure the prevalence of asthma in adults in Malaysia	19	91	90	83	71	88	78	100	92	95	83	86.9	0.609
11	Community health worker driven COPD awareness and screening program. Opportunities and challenges for a National COPD Prevention and Control Program.	6	85	85	85	81	81	84	100	88	93	84	86.7	0.700
12	Care-seeking practices of and barriers to; care-seeking for pneumonia in children aged less than five years in tribal and non-tribal rural areas of Pune district, India	38	92	98	87	83	88	66	93	90	93	76	86.6	0.700
13	Palliative care needs of patients with chronic obstructive pulmonary disease	3	90	89	83	79	90	87	91	79	93	82	86.5	0.700
14	Culturally Tailored School-Based Complex Interventions for Childhood Asthma in Malaysia (cut-asthma): An Implementation Study	8	90	89	91	93	82	80	88	84	93	73	86.2	0.657
15	Community health worker driven COPD awareness and screening program. Opportunities and challenges for a National COPD Prevention and Control Program	13	81	88	83	78	83	80	96	88	97	83	85.7	0.443
16	Developing and pilot-testing the effectiveness of a culturally tailored awareness and supported self-management intervention for patients with COPD	17	83	78	82	88	91	82	95	86	88	83	85.4	0.613
17	Long-term respiratory morbidity (asthma) in children with RSV in the neonatal period- follow up of ANISA Cohort in Sylhet	35	86	72	82	78	71	85	97	88	91	88	83.8	0.487
18	Enhancing access to pulmonary rehabilitation (PR) through implementation research in Bangladesh	11	90	70	88	92	71	81	89	88	93	75	83.5	0.596
19	Evaluation of mobile phone (M- Health) technology for self management in pollen asthma patients in Pakistan	10	88	80	81	88	82	73	90	75	91	86	83.4	0.617
20	Reliability and validity of asthma control test among Chinese, Malays and Indians in Malaysia	5	96	98	74	78	95	62	90	81	91	67	83.2	0.670
21	Trial of pulmonary rehabilitation	25	81	77	83	92	85	72	82	77	93	81	82.2	0.448
22	Feasibility of using m-health technology to counsel caregivers of children under five on prevention against pneumonia and improved care seeking through lady health workers in rural communities	36	77	79	85	90	74	81	92	79	84	80	82.1	0.630
23	Long-term respiratory morbidity (asthma) in children with RSV in the neonatal period- follow up of ANISA cohort in 3 South Asian countries	34	87	72	82	80	63	82	94	79	94	85	81.8	0.535
24	Factors responsible for uncontrolled asthma in a rural community in Southern India	9	82	81	78	73	87	70	98	83	91	73	81.6	0.643
25	Integrating childhood asthma detection and management into the primary health care in rural Pune district	22	83	79	83	85	82	77	85	85	93	64	81.5	0.570
26	Use of telemedicine in management of COPD and asthma in rural areas where medical expertise is not available	16	80	74	79	90	72	78	95	85	91	72	81.5	0.517
27	Exploration of pneumonia-related policy formation and implementation in Pakistan	30	91	89	85	64	83	68	89	81	92	71	81.5	0.526
28	Development of spirometry predictive values for Indian population	1	86	90	77	78	84	72	89	71	91	76	81.4	0.639
29	Feasibility of using family psychoeducation for pneumonia prevention among caregivers of children under five	37	81	79	78	79	87	71	95	88	87	64	80.7	0.509
30	Assessment of ASHA’s (accredited social health activist) workload and its determinants	4	94	93	74	73	88	65	70	84	93	69	80.3	0.648
31	Implementation of bubble CPAP in the management of severe pneumonia in young children in secondary level health facilities	33	93	89	90	82	74	70	87	64	65	89	80.2	0.517
32	Community use of digital auscultation to improve diagnosis of paediatric pneumonia in Sylhet, Bangladesh	26	85	80	81	79	76	81	90	68	79	75	79.4	0.504
33	Prevention, detection and treatment of adult lung disease (with a focus on lung cancer) in a poor, rural population in Tamil Nadu	18	71	60	68	76	84	69	94	85	94	64	76.8	0.474
34	Lung cancer and chronic respiratory disease: Development and pilot testing of an intervention in a southern Indian rural community	14	75	64	76	83	69	69	93	84	90	60	76.4	0.526
35	Documenting pneumonia case management practices in selected communities in Pakistan: a qualitative study	29	83	93	68	63	83	60	84	85	84	56	75.9	0.474
36	Allergen mapping and seasonal exacerbations of asthma in Pakistan	23	78	76	72	75	64	47	83	70	93	61	72.0	0.391
37	Construction of a computational framework to automatically interpret chest x-rays and diagnose pneumonia	28	69	68	64	70	71	75	83	72	72	68	71.2	0.443

*Their research priority scores are shown for each of the 10 priority-setting criteria. The overall research priority score, which provides the basis for the final ranking, is also presented, along with the average expert agreement. Detailed explanations of these measures are provided in references [4,8-11].

## OUTCOMES

**Table 1** provides the final rankings for the 37 proposed research questions that relate to global respiratory health and are of interest to the RESPIRE initiative. Research priority scores for these ideas are shown for each of the 10 priority-setting criteria and they are self-explanatory, as well as the overall research priority score, which is the basis for the final ranking.

Research ideas with the highest scores included proposals to measure the burden of COPD in the four partner countries, along with the development of a protocol for a COPD prevalence study; also, HEAL-ASTHMA studies, a cross-sectional study to determine the prevalence of limited health literacy followed by a quasi-experimental feasibility study of a pictorial action plan for asthma in Malaysia; then, implementation of pulse oximetry as a point-of-care diagnostic in health facilities that provide integrated management of childhood illness; assessing the feasibility of using a well-established tele-consultation facility (Micro-Health Centre -MHC) in management of COPD and Asthma in a resource constrained remote rural area; and studying the perception of under-five pneumonia among caregivers of selected rural and semi urban communities of Pakistan through a mixed-methods study.

Most importantly, all projects received an overall research priority score greater than 70.0, while a typical range of scores in CHNRI exercises is between 27.0-91.0 [4]. This means that the 26 RESPIRE investigators felt that all 35 proposed research ideas were promising, had few apparent shortcomings and were worth doing. However, there were rather strong internal differences in opinions related to 8 out of the 37 proposed projects, as captured by AEA<50.0 (**Table 1**), which meant that some of the proposed ideas were probably more controversial than others.

There are also some specific concerns related to some proposed research questions in relation to particular criteria. The criteria around which most concerns were raised, as captured by the criterion-specific research score <70.0, were sustainability (in 9 cases), scalability (in 7 cases) and effectiveness (in 3 cases; see **Table 1**).

## CONCLUSIONS

The CHNRI exercise worked very well within the RESPIRE initiative and gave support to all proposed research ideas, while highlighting some specific topics that warranted further discussions. It served the purpose of quickly assessing strengths and weaknesses of the proposed research ideas that we believed could help reduce respiratory disease burden in partner countries by 2025. This was in line with recommendations from RESPIRE's International Steering Committee, which recommended that prioritisation takes place to allow for partner input into funding allocation decisions.

The CHNRI process provided a useful complementary insight to that provided by the peer-review process into perceived strengths and weaknesses of each proposed research project and PhD project. It informed the prioritisation of funding for large RESPIRE projects, smaller projects and PhD projects, in addition to the Panel discussion in Dhaka in February 2018 and the external peer review. Its results showed a rather narrow range in the overall research priority scores, ie, between 71.2 and 91.9. We believe that this is primarily a result of very well explained context and rules of the CHNRI project to all participants in the exercise during the first RESPIRE meeting in Edinburgh in June 2017. This led to an initial selection of a relatively small number of research ideas, all of which arose from the reality of the contexts of the partner countries as real needs. All of the proposed projects had a reasonable chance of implementation and success in generating new knowledge, and this was reflected in the overall scores. Typical CHNRI exercises can usually have up to 10 times more research ideas proposed by a much larger groups of researchers, and many of their ideas may well be quite speculative and risky, leading to a much wider dispersion of the overall research priority scores.

The process was not free from some limitations. Primarily, scorers in the CHNRI exercise were not independent from the experts who set the topics. Also, in a sample size of 37 larger sampling errors can be expected. After the exercise was conducted, some of the scorers commented that more detail could have been provided to some of the proposed ideas, as this could have affected their scores. Some ideas were changing over the period of the CHNRI process as they were being developed. Therefore, it is possible that some scorers may have missed the most recent update on some ideas (eg, if they couldn't attend the latest RESPIRE teleconference). There were also comments from the scorers that a subsequent, larger exercise could be considered. Such exercise would include more ideas, scorers and a longer time frame. Future exercises could also be improved if they considered not only opinions from scorers, but also their confidence in their opinions. Such an approach has been shown to increase the validity of exercises which use crowdsourcing to derive collective opinion [12-14].
